# A Novel Chimpanzee Adenovirus Vector with Low Human Seroprevalence: Improved Systems for Vector Derivation and Comparative Immunogenicity

**DOI:** 10.1371/journal.pone.0040385

**Published:** 2012-07-13

**Authors:** Matthew D. J. Dicks, Alexandra J. Spencer, Nick J. Edwards, Göran Wadell, Kalifa Bojang, Sarah C. Gilbert, Adrian V. S. Hill, Matthew G. Cottingham

**Affiliations:** 1 The Jenner Institute, University of Oxford, Oxford, United Kingdom; 2 Department of Clinical Microbiology, University of Umeå, Umeå, Sweden; 3 Medical Research Council Laboratories, Fajara, The Gambia; French National Centre for Scientific Research, France

## Abstract

Recombinant adenoviruses are among the most promising tools for vaccine antigen delivery. Recently, the development of new vectors has focused on serotypes to which the human population is less exposed in order to circumvent pre-existing anti vector immunity. This study describes the derivation of a new vaccine vector based on a chimpanzee adenovirus, Y25, together with a comparative assessment of its potential to elicit transgene product specific immune responses in mice. The vector was constructed in a bacterial artificial chromosome to facilitate genetic manipulation of genomic clones. In order to conduct a fair head-to-head immunological comparison of multiple adenoviral vectors, we optimised a method for accurate determination of infectious titre, since this parameter exhibits profound natural variability and can confound immunogenicity studies when doses are based on viral particle estimation. Cellular immunogenicity of recombinant E1 E3-deleted vector ChAdY25 was comparable to that of other species E derived chimpanzee adenovirus vectors including ChAd63, the first simian adenovirus vector to enter clinical trials in humans. Furthermore, the prevalence of virus neutralizing antibodies (titre >1∶200) against ChAdY25 in serum samples collected from two human populations in the UK and Gambia was particularly low compared to published data for other chimpanzee adenoviruses. These findings support the continued development of new chimpanzee adenovirus vectors, including ChAdY25, for clinical use.

## Introduction

Recombinant adenoviruses were originally developed for gene therapy [1], but the strong and sustained transgene-specific immune responses elicited by these delivery agents, together with their broad tissue tropism, has prompted their use as vaccine vectors [2]. Deletion of a single transcriptional unit, E1, renders the virus replication incompetent, reducing the potential for side effects in clinical applications. Deletion of a second unit, E3, increases the insert capacity to 8 kb, allowing flexibility in antigen design, and does not affect growth in an E1-complementing cell line. The first generation of vaccine vectors based on human adenovirus type 5 (HAdV-5), the most widely studied adenoviral serotype, showed poor efficacy in HIV-1 clinical trials despite encouraging pre-clinical data [3], [4]. A large proportion of human adults possess significant titres of neutralising antibodies to common human serotypes such as HAdV-2 and HAdV-5. Neutralising antibodies have the potential to reduce the potency of viral vector vaccines by inhibiting vector mediated delivery of the encoded transgene. Pre-existing anti vector immunity has since been addressed through the development of new vectors based on serotypes to which the human population is less exposed, including those of chimpanzee origin [5], [6], [7]. Chimpanzee adenoviral vectors have been shown to be highly immunogenic in animal models [8], [9] and recently in clinical malaria vaccine trials [10], [11].

Adenovirus vectored vaccines have been widely used in early stage clinical trials targeting a range of diseases including malaria, HIV, influenza, hepatitis C, and cancer [12]. However, despite the large number of clinical trials, to date only a handful of serotypes (HAdV-5, HAdV-6, HAdV-35 and two chimpanzee adenovirus serotypes ChAd63 and ChAd3) have been assessed as vaccine vectors in humans. There is a need for development of new vectors for clinical application, both to assess the utility of different serotypes and to enable deployment of multiple vaccines within a single population, since anti-vector immunity after immunisation may limit the efficacy of a second immunisation with the same vector [13]. New vectors will need to be based on viruses that have low seroprevalence in humans, and are able to elicit robust transgene product specific immune responses.

Here we describe the development of a new adenoviral vector based on a chimpanzee adenoviral isolate Y25 [14], the genomic sequence of which we report here. We used a bacterial artificial chromosome (BAC) system to generate a molecular clone of the virus, to which precise genetic modifications were made through BAC recombineering [15]. The same method was also applied to re-derivation of a BAC clone of SAdV-25 (also known as AdC68 and Pan 9 [5]). This approach offers superior flexibility and speed of vector modification over previously reported methods, as well as improved genetic stability [16]. We demonstrate experimentally that the infectivity of adenoviral vector preparations is the principal determinant of immunogenicity (rather than viral particle count) [7] and use a single cell infectivity assay to assess infectious titer more accurately and conveniently than the traditional plaque assay or endpoint dilution methods. Using this improved strategy, we show, in both single immunisation and prime boost regimens, that cellular immunogenicity of new vector ChAdY25 in mice is equivalent to that of existing chimpanzee adenoviral vectors. Our comparison includes ChAd63, previously shown to be highly immunogenic in man [10], [11]. The seroprevalence of vector neutralising antibodies against Y25 was found to be similar to or lower than previously reported for other chimpanzee adenoviruses in the British and Gambian human populations tested. We therefore propose that ChAdY25 also has the potential to be an efficacious vaccine vector in human clinical trials.

## Materials and Methods

### Ethics Statement

All mouse procedures were performed in accordance with the terms of the UK Animals (Scientific Procedures) Act Project Licence (PPL 30/2414) and were approved by the University of Oxford Animal Care and Ethical Review Committee. For clinical samples, all human volunteers gave written informed consent prior to participation and the studies were conducted according to the principles of the Declaration of Helsinki and in accordance with Good Clinical Practice (GCP). UK samples were obtained from trials MAL34 (NCT00890760) and VAC33 (NCT00890019). For these trials, approvals were granted by the Oxfordshire Research Ethics Committee (OXREC), the Medicines and Healthcare products Regulatory Agency (MHRA) and the Gene Therapy Advisory Committee (GTAC). Gambian samples were obtained from trial VAC41 (NCT01373879) with approval from Oxford Tropical Research Ethics Committee and the State Services Commission (SSC).

### Viruses and Cells

The wild type chimpanzee adenovirus isolate Y25 was originally obtained from William Hillis, John Hopkins University of Medicine. The virus was passaged in HEK293A cells (Invitrogen, Cat. R705-07) and purified by CsCl gradient ultracentrifugation as previously described [17]. Viral DNA was phenol extracted for genomic sequencing and cloning. An AdC68 vector (also known as Pan9 [18]) was independently rederived from genomic DNA isolated from wild-type SAdV-25 virus obtained from ATCC using a similar method to that described here for Y25 (Y. Roshorm *et al.*, manuscript in preparation). The ChAd63 vector was provided by Okairòs AG, Italy.

### Genome Sequencing and Phylogenetic Analysis

Sequencing of the Y25 viral genome was performed by Eurofins MWG Operon AG using GS SLX Titanium Series technology. Previously reported sequences were obtained from Genbank, accession numbers as follows:

ChAd3 CS479276; ChAd63 CS479277; HAdV-5 AC_000008; SAdV-22 AY530876; SAdV-25 AF394196, HAdV-1 AF534906; HAdV-2 J01917; HAdV-4 AY458656; HAdV-6 FJ349096; HAdV-12 X73487; HAdV-16 AY601636;; HAdV-30 DQ149628; HAdV-10 AB369368, AB330091; HAdV-17 HQ910407; HAdV-9 AJ854486; HAdV-37 AB448776; HAdV-8 AB448767; HAdV-7 AB243119, AB243118; HAdV-11 AC_000015; HAdV-21 AY601633; HAdV-34 AY737797; HAdV-35 AC_000019; HAdV-40 NC_001454; HAdV-41 DQ315364; SAdV-21 AC_000010; SAdV-23 AY530877; SAdV-24 AY530878; HAdV-3 DQ086466; HAdV-18 GU191019; HAdV-31 AM749299; HAdV-19 AB448774; SAdV-25.2 FJ025918; SAdV-30 FJ025920; SAdV-26 FJ025923; SAdV-38 FJ025922; SAdV-39 FJ025924; SAdV-36 FJ025917; SAdV-37.1 FJ025921; HAdV-14 AY803294; SAdV-27.1 FJ025909; SAdV-28.1 FJ025914; SAdV-33 FJ025908; SAdV-35.1 FJ025912; SAdV-31.1 FJ025906; SAdV-34 FJ025905; SAdV-40.1 FJ025907; SAdV-3 NC_006144;Y25 JN254802.

Sequence alignment was performed with DNAStar Megalign software using the ClustalW algorithm [19]. Phylogenetic and bootstrap analyses were performed utilising the neighbour joining method with 100 bootstrap reconstructions for each gene. Trees were modified in FigTree v1.3.1[20].

### BAC Rescue of Y25 Genome

To generate the pBAC ‘rescue’ vector, two homology flanks from the left end of the Y25 genome (Left Flank I, LFI and Left Flank II, LFII) and one from the right end of the genome (Right Flank, RF) were amplified using Phusion HF (Finnzymes) from Y25 wild type DNA and assembled together using conventional cloning (Table S1). For single step gap repair insertion of the Y25 genome into the BAC, BJ5183 electrocompetent *E. coli* cells (Stratagene) were co-transformed with 20 ng BAC vector and 500 ng Y25 genomic DNA and selected using the chloramphenicol resistance marker on pBACe3.6. Resulting colonies were screened by colony PCR for insertion of the genome at both flanks, and for deletion of the E1 region; a consequence of homologous recombination between BAC and genome at LFII instead of LFI. E1 deleted clones were selected, and genome integrity was checked by restriction digest.

### Manipulation of E1, E3, E4 Regions by Recombineering

Galactokinase (*GalK*) based recombineering was performed as described in Warming *et al* [15]. *GalK* replacement of the E3 region was performed using primers E3delLH and E3delRH (Table S1). The amplified *GalK* gene in this cassette was flanked by *Pac*I sites, enabling *GalK* deletion by restriction digestion and re-circularization rather than counterselection to leave a unique *Pac*I site at E3. A unique *Asi*SI site at the site of E1 deletion was subsequently used to insert a modified *attR1 attR2* Gateway® cassette (Invitrogen) for site specific directional insertion of recombinant antigens into E1. Preparation of the TIP model antigen has been described previously [21]. Finally, the E4 region was modified to optimise growth rate and yield in human cell lines. Two *GalK* insertions were made, to delete either the entire native E4 region excluding the E4 promoter (using primers E4delLH/E4wholedelRH), or only open reading frames (Orfs) 4, 6 and 7 (using primers E4delLH/E4Orf4delRH). Subsequent counterselection replacements of *GalK* with regions of the HAdV-5 E4 genome were performed using PCR products amplified from a HAdV-5 genomic clone pAd-PL DEST (Invitrogen). Negative selection against *GalK* using 2-deoxygalactose was performed as described in Warming *et al* [15].

### Vector Propagation and Purification

Plasmid or BAC DNA from recombinant molecular clones were linearised with *Pme*I (ChAdY25, AdC68) or *Pac*I (HAdV-5, ChAd63) to release the left and right viral inverted terminal repeats (ITRs). Linearised DNA (6 μg) was transfected onto Human Embryonic Kidney (HEK) 293A cells (Invitrogen) in 6-well plates using Lipofectamine 2000 reagent (Invitrogen). After cytopathic effect (CPE) was observed, the cells were harvested, subjected to three cycles of freeze-thaw, and the virus amplified further in 293 cells. After passage through a T150 flask the virus was titered (see *Vector Titration*) and used to infect a Hyperflask (Corning Inc.) at a multiplicity of infection (MOI) of 5. Virus was harvested from infected cells after 48 hours and purified by CsCl gradient ultracentrifugation according to standard practice. Purified virus was dialysed against sucrose storage buffer (10 mM Tris, 7.5% w/v sucrose. pH 7.8) and stored at −80°C.

### Vector Titration

For infectivity assays, virus for titration was serially diluted in DMEM supplemented with 10% FCS and 50 μl of inoculum was added per well of a 96 well plate containing sub-confluent HEK293A cells. After 24 hours a further 50 μl media was added to cells, and after 48 hours infectivity was assessed. For GFP expressing viruses, the GFP positive cells were visualised directly and counted by fluorescent microscopy. For marker-less viruses, an anti-hexon immunostaining assay based on the QuickTiter™ Adenovirus Titer Immunoassay kit (Cell Biolabs Inc) was used [22]. Cells were fixed at −20°C in methanol, before the plates were blocked with 1% bovine serum albumin (BSA) in phosphate buffered saline (PBS). Adenovirus hexon protein was stained by addition of 50 ul per well primary mouse anti-hexon polyclonal antibody (Cell Biolabs Inc.) followed by 50 ul secondary anti mouse horseradish peroxidise (HRP) conjugated antibody. Staining was developed by addition of 3,3′ diaminobenzidine (DAB) substrate, and positive brown stained cells in the wells were counted by light microscopy. For detection of Matrix Protein 1 (M1) antigen an anti-M1 monoclonal antibody (Abcam, Cambridge, UK) was used. Viral particles were measured by spectrophotometry as described previously [23].

### Mice and Immunisation

Female Balb/c mice (Harlan UK Limited) were immunised intramuscularly at 6–7 weeks of age according to UK legislation. 50 μl viral vector in PBS was injected into the tibialis muscle of a single hind limb of each animal.

### Ex vivo IFN-γ Spleen ELISpot

Spleens were harvested at the relevant time-point post immunisation and *ex vivo* IFN-γ ELISpot was performed as described previously [21]. Cells were re-stimulated for 18–20 h with Pb9 peptide (CD8^+^ epitope from *Plasmodium berghei* circumsporozoite protein SYIPSAEKI) [24] or P15 peptide (CD4^+^ epitope from *Mycobacterium tuberculosis* Ag85A MTFLTSELPGWLQANRHVKPT) [25] at a final concentration of 1 µg/mL.

### Blood Ex-vivo Intracellular Cytokine Staining (ICS)

Peripheral blood mononuclear cells were lysed with ACK to remove RBCS prior to re-stimulation for 5 h with 1 µg/mL Pb9 in the presence of 2 µg/mL Golgi-Plug (BD). Cells were surface stained with anti-CD8 PerCPCy5.5 conjugate and anti-CD4 APC-Alexa-780 conjugate prior to fixation in 10% neutral buffered formalin (Sigma). Intracellular cytokine staining was performed using APC-conjugated anti-IFN-γ diluted in Cytoperm (BD). All antibodies were obtained from eBiosciences. Data were acquired on a CyAn flow cytometer (Dako) and analysed using FlowJo (Treestar). Statistical analyses were performed using Prism (GraphPad Software, Inc.).

### Anti- Vector Neutralising Antibody Assay

Pre vaccination human serum samples were stored at −80°C and heat inactivated at 56°C for 90 min prior to use. The assay was performed as described previously [6], with the following modifications. Briefly, GripTite 293 MSR cells (Invitrogen, catalogue no. R795-07, cultured as per manufacturer’s instructions) seeded the previous day at 3×10^4^ cells per well were infected with a 1∶1 mixture of test sera dilutions and ChAdY25-E–SEAP (secreted alkaline phosphatase) recombinant vector. Recombinant adenoviruses were incubated with five serial dilutions of serum in FBS–DMEM (phenol red free; GibcoBRL catalog no. 31053-028) before infection. The final serum dilutions were 1∶18, 1∶72, 1∶288, 1∶1152, 1∶4608; each serum sample was tested in duplicate. Supernatants were collected and assayed for SEAP concentration using CSPD (Tropix PhosphaLite Chemiluminescent Assay Kit, Applied Biosystems UK) according to the manufacturer’s instructions. Luminescence intensity was measured using a Varioskan flash luminometer (Thermo Scientific). Neutralization titers were defined as the serum dilution required to reduce SEAP concentration by 50% compared to wells infected with virus alone. Neutralization titer was calculated by linear interpolation of adjacent values.

## Results

### Y25 Clusters Phylogenetically with Human and Chimpanzee Adenoviruses Belonging to the Species *Human Adenovirus E*

**Figure 1:**
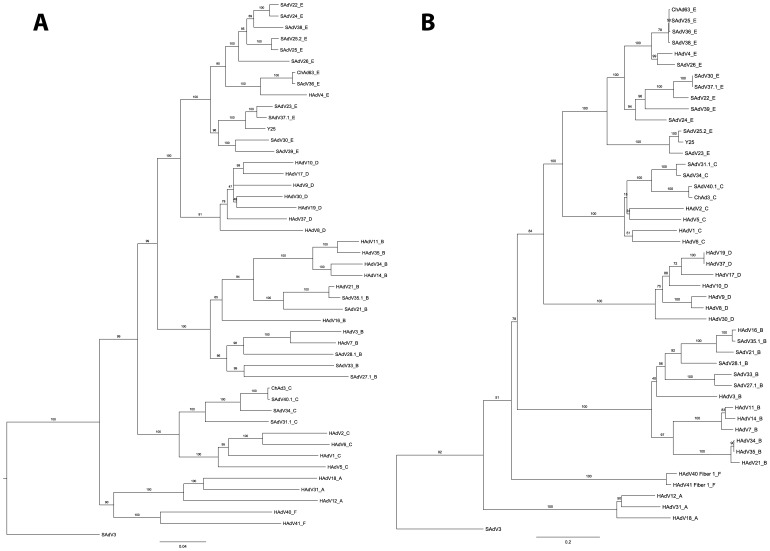
Phylogenetic trees based on alignment of nucleotide sequences of (A) the hexon protein and (B) the fiber protein of different human (HAdV-) and chimpanzee (SAdV-) adenovirus serotypes including Y25. Note that SAdV-3 (species *Simian adenovirus A*) is included here as an outgroup, having been derived from a Rhesus Macaque. Clustering of sequences into each of the species *Human adenovirus A-F* is indicated below. Bootstrap values for branches indicated.

The chimpanzee adenovirus isolate Y25, was first described by Hillis *et al* [14]. We obtained and propagated the isolated virus and sequenced the viral genome (GenBank accession no. JN254802). Previous literature on this isolate is scarce, but the genome sequence data has confirmed early serological indications that this adenovirus is related to the *Human adenovirus E* virus, HAdV-4 [26]. Simian adenoviruses (SAdV) isolated from great apes are not phylogenetically distinct from human adenoviruses (HAdV), and group together into the same viral species (*Human adenovirus B, C and E*) [27]. Although HAdV-4 is the sole representative of *Human adenovirus E* derived from humans, many of the chimpanzee adenoviruses group phylogenetically within species E, including vaccine vector candidates ChAd63, AdC68 (SAdV-25), AdC7 (SAdV-24) and AdC6 (SAdV-23) [5], [18], [28], [29]. Phylogenetic trees were constructed based on the DNA sequences of hexon and fiber proteins since these are the major surface-exposed capsid components and are believed to be the primary determinants of vector tropism and serum neutralisation (Figure 1). The phylogenetic analysis indicates that chimpanzee adenovirus Y25 also groups with *Human adenovirus E* viruses. A SAdV isolated from a rhesus macaque (SAdV-3) does not group phylogenetically with the human and chimpanzee viruses, and is included as an outgroup. Trees based upon the viral DNA polymerase (commonly used to classify adenovirus species), and complete genome sequences also group Y25 in *Human adenovirus E* (data not shown). Interestingly, both hexon and fiber DNA sequences from Y25 and SAdV-23 appear to cluster separately from other *Human adenovirus E* members, in agreement with early serological data suggesting that Y25 and SAdV-23 (CV-32) are more closely related to each other than to their other species E counterparts [26]. In order to assess cross-neutralization of Y25 by another chimpanzee adenovirus belonging to *Human adenovirus E*, we used serum from a recent Phase I clinical trial in which volunteers vaccinated with a recombinant ChAd63 malaria vaccine developed extremely high neutralization titers against ChAd63 (>1∶1,000) [10]. Serum from these subjects did not neutralise Y25 (data not shown), supporting the phylogenetic evidence that Y25 is a distinct serotype from ChAd63, despite being a member of the same species.

### Development of ChAdY25 Vaccine Vector

**Figure 2:**
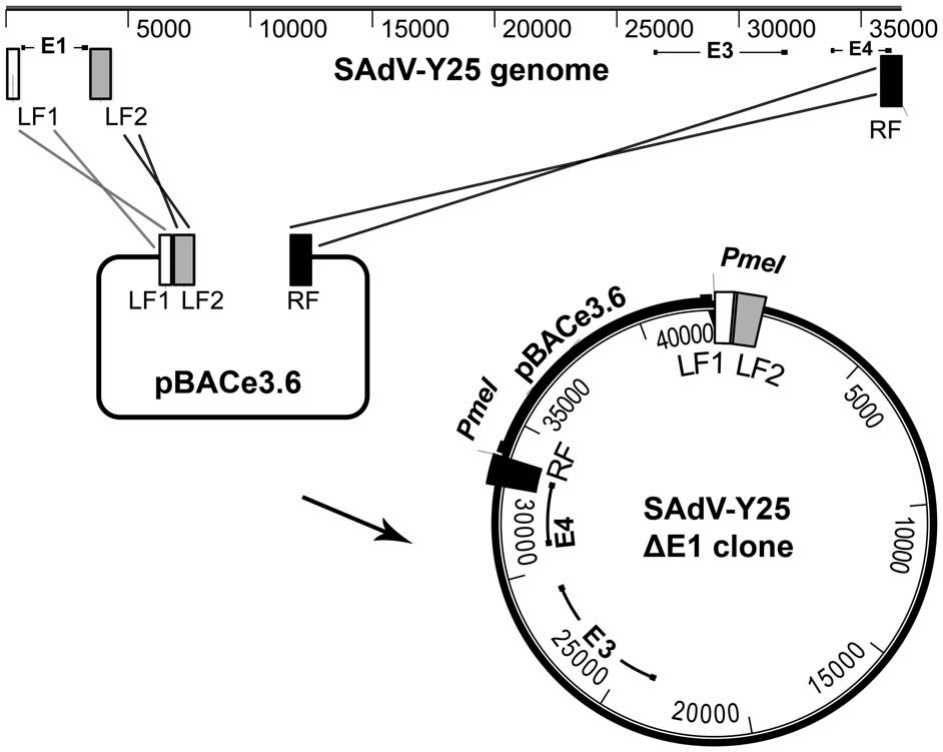
Generation of a molecular clone of Y25 by gap repair insertion of Y25 genomic DNA into the pBAC ‘rescue vector’. Recombination between BAC vector and the left side of the viral genome can either occur at LFI such that the E1 region is included in the resulting BAC clone, or more desirably at LFII such that E1 is deleted.

To generate a molecular clone of the Y25 genome, a BAC gap repair vector was constructed containing PCR-amplified regions of homology to the left and right flanks of the viral genome as described in Chartier *et al* [30] (Figure 2). In our study, an extra homology flank downstream of the adenovirus E1 region was included to enable deletion of E1 and placement of a unique restriction site at the E1 locus, concomitant with genomic insertion into the BAC. The E1 region is essential for viral replication, hence the ability to delete E1 at this stage renders the new vector immediately replication incompetent. Replication incompetent (E1-deleted) clones were successfully identified by PCR screening, and transfection into E1 complementing HEK293 cells [31] confirmed the ability of all candidate clones to generate infectious virions.

Since the viral genome had been incorporated into a BAC rather than a plasmid vector we were able to efficiently introduce further genetic modifications through recombineering, instead of relying on traditional restriction cloning. This technique, based on the lambda phage Red recombination function, has many advantages over restriction cloning: there is no dependence on location of specific recognition sites allowing flexible and seamless introduction and deletion of genetic sequence. This approach has previously been implemented to perform precise modification to the HAdV-5 genome [32], [33]. We utilized the galactokinase (*GalK*) recombineering system in SW102 *E. coli* for both positive and negative selection of recombinant clones [15]. Using this technique the non-essential adenovirus E3 region was deleted, thereby increasing the insert capacity of the new vector by approximately 5 kb. The new E1/E3 deleted vector was termed ChAdY25. Insertion of recombinant antigens at the E1 locus was performed using Gateway® site specific recombination technology (Invitrogen).

### Optimisation of Simian Adenoviral Vector Growth and Yield

**Figure 3:**
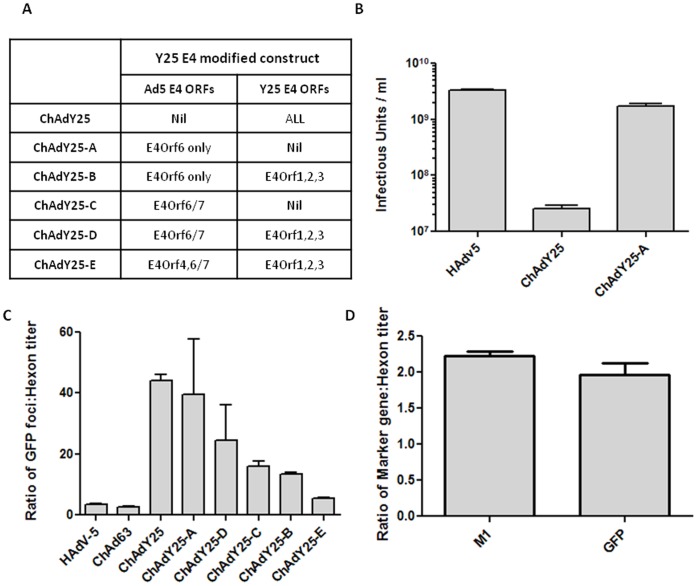
Modification of the Y25 adenovirus E4 region to increase yield and hexon expression for titration. (**A**) Modified Y25 vectors labelled ChAdY25-A to –E. (**B**) Virus yield of Y25 based vectors expressing GFP before and after *E4Orf6* replacement. HEK293 cells were infected at a multiplicity of infection of 10 ifu/cell and incubated at 37°C for 48hours before harvesting. Infectious titer of the harvested material was measured by quantifying GFP positive foci 48 hrs post infection. (**C**) Ratio of GFP titer to anti-hexon titer for different Y25 E4 modified vectors expressing the TIPeGFP antigen. Titers assessed 48 hrs post infection. (**D**) Ratio of transgene: hexon expression for ChAdY25-E based vectors expressing TIPeGFP and Influenza Matrix protein 1 (M1) recombinant transgenes. All data is representative of at least two independent experiments. Error bars show mean and SEM.

The first generation E1/E3 deleted ChAdY25 vectors grew inefficiently in HEK293 cells, and viral yield was approximately two logs lower than for HAdV-5 based vectors (Figure 3B). It has previously been shown that modification of the adenovirus E4 region is necessary for the efficient propagation of some non-HAdV-5 based vectors in HEK293 cells [34]. Although HEK293 cells do support propagation of adenoviruses from other serotypes, yield is often lower and it has been speculated that this is due to a suboptimal interaction between the HAdV-5 E1 proteins expressed by the cell line and vector-encoded E4 gene products required for replication. Of particular importance is the product of *E4Orf6*, a multifunctional protein implicated in late viral mRNA splicing, selective export of viral mRNA, viral DNA synthesis, and inhibition of apoptosis. The function of E4Orf6 is dependent on an interaction with the E1B-55K protein. We therefore generated a series of five ChAdY25 vectors with variant E4 loci, incorporating different regions of the E4 locus from HAdV-5, referred to as ChAdY25-A to -E (Figure 3A). To our knowledge, previously reported E4 modifications to non HAdV-5 recombinant vectors have concerned the introduction solely of *E4Orf6* from HAdV-5. In the case of ChAd63, the entire native E4 locus was deleted prior to insertion of the HAdV-5 *E4Orf6* gene [35]. As expected, a similarly modified virus, ChAdY25-A significantly improved virus yield in HEK293 cells (Figure 3B). This improvement in yield enabled production of enough virus to perform comparative immunogenicity studies using ChAdY25-A.

### Infectious Units and not Viral Particles Correlate with Immunogenicity

**Figure 4:**
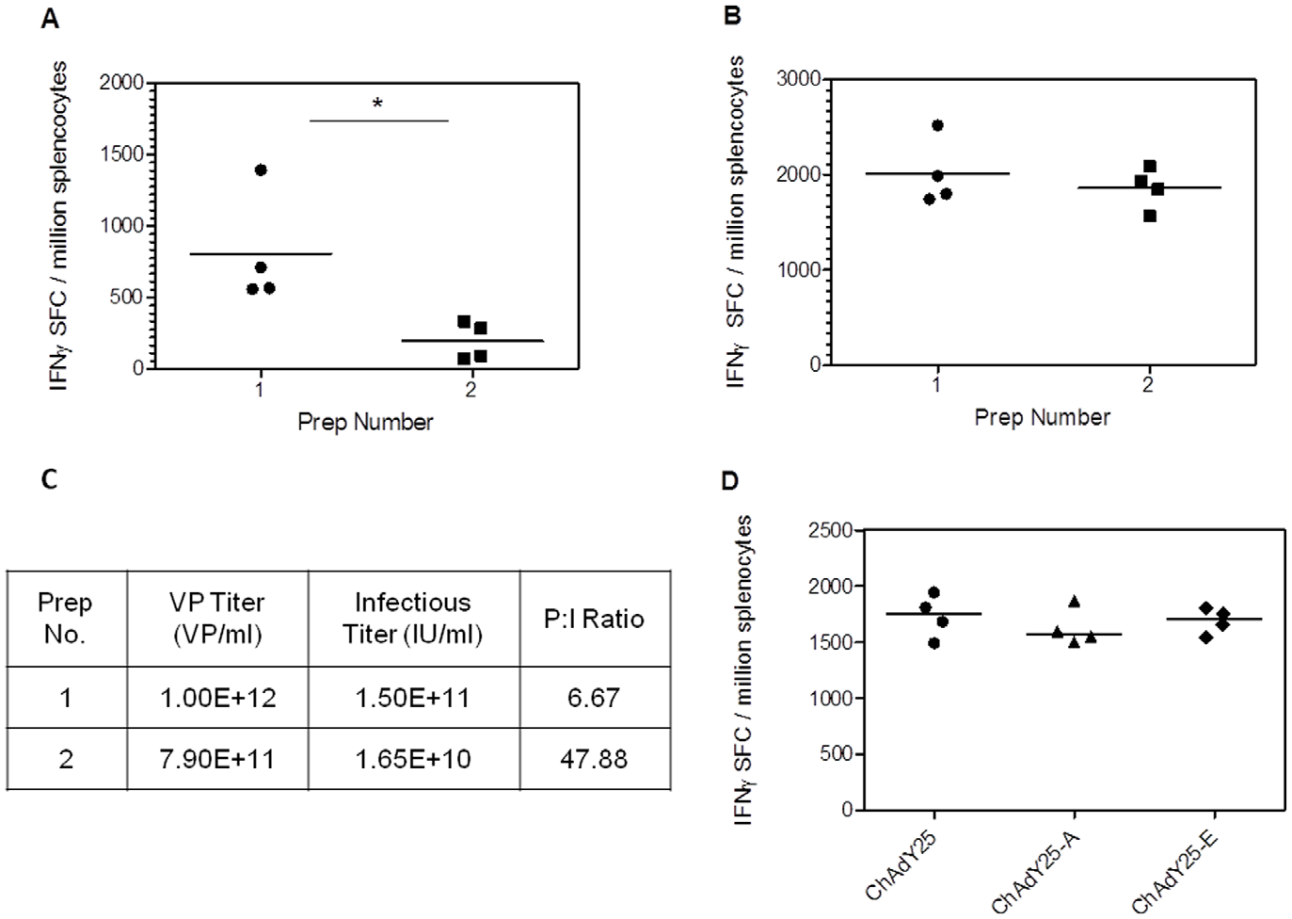
Infectivity of recombinant adenovirus vector preparations can affect immunogenicity. (**A–C**) IFN-γ Spleen ELISpot responses after immunisation with HAdV-5 TIPeGFP doses based on viral particles (VP) or infectious units (IU). Balb/c mice were immunised intramuscularly with either (**A**) 10^7^ viral particles or (**B**) 10^7^ infectious units of one of two independent preparations of HAdV-5 TIPeGFP. Responses to CD8^+^ epitope Pb9 were assayed two weeks post immunisation. Asterisk indicates p<0.05. (**C**) Titers per ml and particle to infectious unit (P:I) ratios of the two CsCl-band purified vector preparations. (**D**) Immunogenicity of TIPeGFP expressing Y25 based vectors with different E4 modifications is comparable despite the vectors having different P:I ratios. Balb/c mice were immunised intramuscularly with 10^8^ infectious units of Y25 vectors with either i) a native E4 locus (ChAdY25) ii) Ad5E4Orf6 expressed at E4 (ChAdY25-A) or iii) the clinical ChAdY25-E vector. IFN-γ Spleen ELISpot was performed as in A-C. P:I ratios of vector preparations were as follows; ChAdY25 **131**, ChAdY25-A **17.4**, ChAdY25-E **13.4**.

Encouraging pre-clinical immunogenicity in mice with the ChAd63 vector [29] has translated into induction of transgene specific T cell responses to unprecedented frequencies in recent human clinical trials [11]. An aim of this study was to determine whether comparable immunogenicity to ChAd63 could be obtained in mice using ChAdY25. The vast majority of immunogenicity studies of adenoviral vectors to date have based immunisation doses on viral particle number (a spectrophotometric measurement of absorbance at 260 nm or real-time PCR to quantify viral DNA) [8], [9], [36], [37]. While rapid and straightforward, this measurement is not only susceptible to nucleic acid contamination, but provides no information on the ability of viral particles in the vector preparation to infect cells – which is required for synthesis of the recombinant antigen protein and hence for immunogenicity. It has previously been shown that the ratio of viral particles to infectious units (often termed P:I ratio) can vary over several orders of magnitude between virus preparations, and significantly affects immunogenicity [7]. We have confirmed this observation by generating two independent cesium chloride purified preparations of HAdV-5, one of which was harvested at a later time post-infection in order to generate an increased P:I ratio. A vector expressing a model antigen, TIPeGFP, was used in order to allow quantification of viral infectious units by GFP fluorescence microscopy. TIP is an epitope string consisting of murine T cell epitopes including *Pb*9 (a dominant H2-K^d^ restricted CD8^+^ T cell epitope from the circumsporozoite protein of *Plasmodium berghei*) and P15 (a CD4^+^ T cell H2^d^ restricted epitope from *M. tuberculosis* antigen 85A) and is fused to the N-terminus of enhanced GFP [38]. The divergent P:I ratios were confirmed upon determination of viral particle and infectious titres (Figure 4C). As expected, differences in the P:I ratio between preparations significantly affected the immunogenicity of the vector when doses were calculated using viral particle counts, but no such bias was observed when infectious titres were used (Figure 4A, 4B, and 4C). Thus, we conclude that, despite the widespread use of viral particle estimation, comparisons of adenoviral vector immunogenicity are not adequately controlled unless the viral infectivity has been accurately determined. In order to conduct a fair comparison of the immunogenicity of ChAdY25-A with other vectors, we therefore decided to base doses on infectious units.

**Figure 5:**
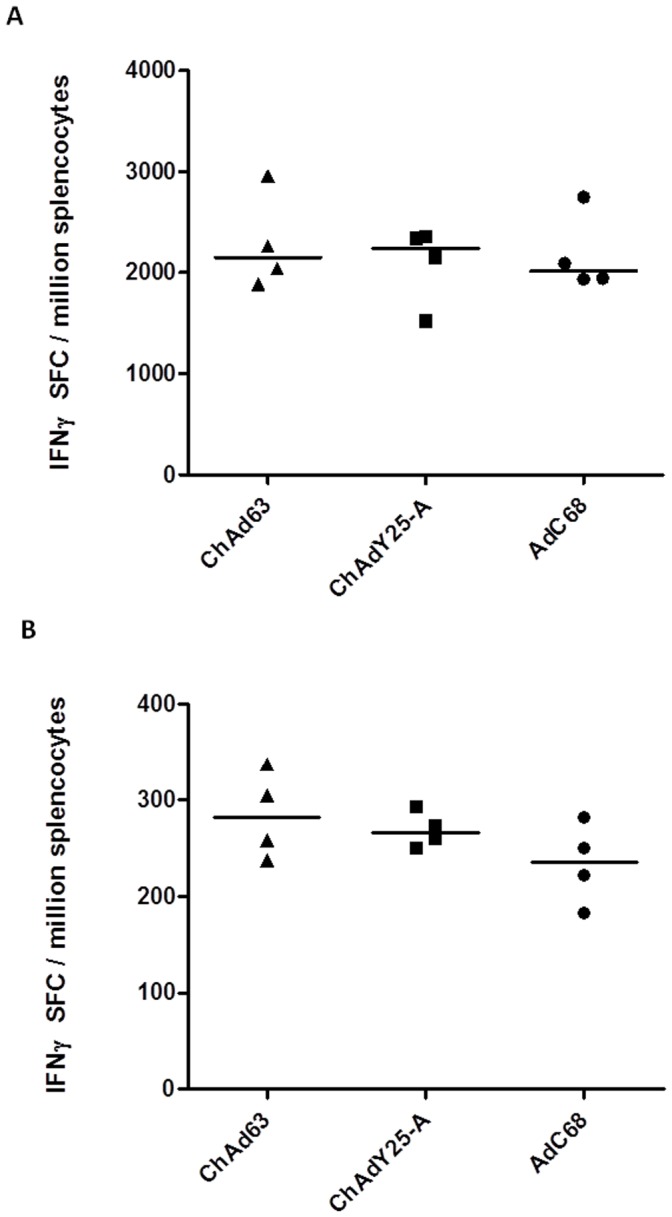
Cellular immunogenicity of ChAdY25 is robust and comparable to current chimpanzee adenovirus vectors AdC68 and AdC63. Balb/c mice were immunised with 10^9^ infectious units of AdC68TIPeGFP, AdC63TIPeGFP or ChAdY25-A TIPeGFP. Two weeks post immunisation, spleen immunogenicity against (**A)** dominant CD8^+^ epitope (Pb9) and (**B**) CD4^+^ epitope (P15) was assessed by IFNγ ELISpot. P:I ratios of vector preparations were as follows; ChAd63 **24.9**, ChAdY25-A **17.41**, AdC68 **31.6**.

The results shown in Figure 4 do not indicate that excess non-infectious viral particles have any adjuvant-like effect on cellular immunogenicity when doses of infectious virus are equalized. We nevertheless wished to minimize as far as possible the variables that might bias or introduce artefacts into comparative immunogenicity studies. As described in Materials and Methods, we therefore used a fixed multiplicity of infection, based on an infectious unit titration, for the final stage of viral amplification prior to purification. This allowed preparation of stocks of different viruses with very similar total yields and P:I ratios (P:I ratios ranged from 17.4 to 31.6 for vectors used in comparative studies – see Figure 5).

### Immunogenicity of ChAdY25 Vaccine Vector is Equivalent to that of AdC68 and ChAd63

Immunogenicity of ChAdY25-A in comparison to the E1/E3 deleted chimpanzee adenovirus vectors ChAd63 and AdC68 was assessed using the model antigen TIPeGFP. T cell responses to epitopes Pb9 and P15 were determined by IFNγ ELIspot. The three vectors had similar P:I ratios (Figure 5) and E4 modifications (i.e. deletion of the native E4 and replacement with HAdV-5 *E4Orf6*, Figure 3C construct ChAdY25-A). Immunisation dose was based on infectious titer as determined by enumeration of single GFP^+^293 cells (see Methods). Immunogenicity was assessed two weeks post immunisation, at the peak of the effector CD8^+^ T cell response [13]. In a head-to-head comparison at a dose of 10^9^ GFP infectious units, the frequencies of Pb9 specific CD8^+^ T cells and p15 specific CD4^+^ T cells induced by ChAdY25-A were equivalent to those induced by AdC68 or ChAd63 (Figure 5).

**Figure 6:**
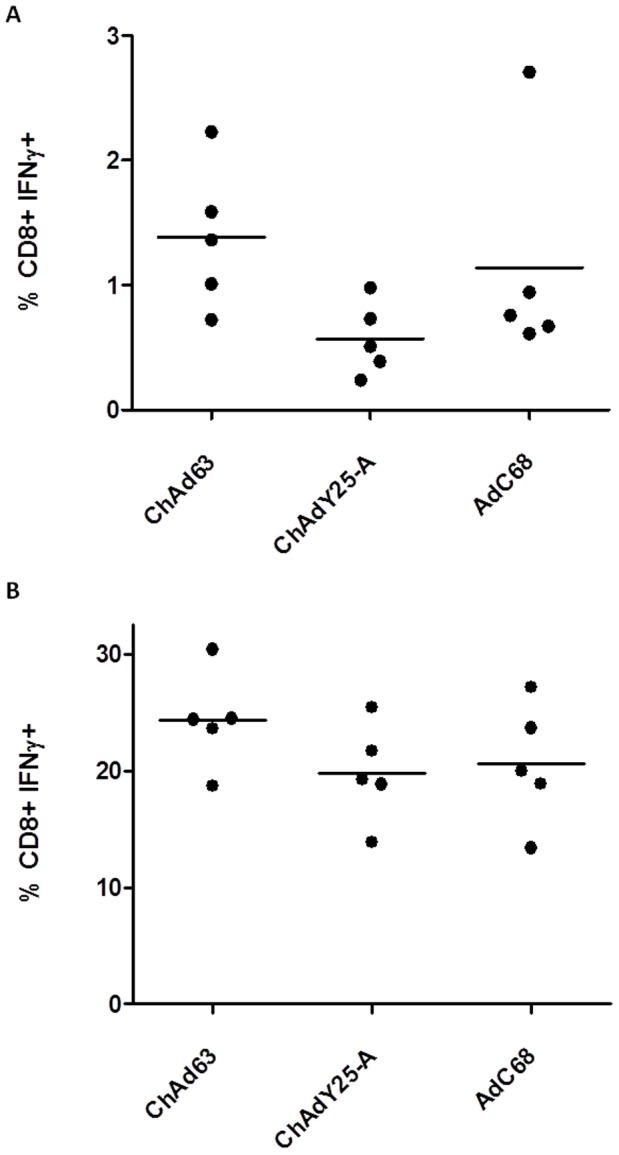
Immunogenicity of SAdV vectors boosted by MVA. Balb/c mice were immunised with 10^6^ infectious units of AdC68-TIPeGFP, AdvY25TIPeGFP, or ChAd63 TIPeGFP. After 56 days post prime, mice were boosted with 10^6^ pfu MVA-TIPeGFP. Blood was collected **A**, 55 post prime and **B**, 7 days post boost. Blood IFNγ^+^ CD8^+^ T cell responses were measured by intracellular cytokine staining (ICS) after stimulation with Pb9 peptide. No statistical significance was found between any of the groups by one way ANOVA.

In both pre-clinical and clinical settings, adenovirus vectors have been shown to act as highly effective priming agents in the context of heterologous prime boost immunisation regimens [11], [39], [40]. Indeed, for many clinical applications, a single adenovirus vector immunisation may be insufficient for protective immunity in humans. Modified vaccinia virus Ankara (MVA) vectors in particular have shown a remarkable capacity to boost adeno-vector induced responses both in pre clinical models, and in human clinical trials [29], [41], [42]. To assess the three chimpanzee vectors in a prime boost regimen, mice were immunised with 10^6^ GFP infectious units of each adenovirus expressing TIPeGFP followed eight weeks later with a boosting immunisation of recombinant MVA encoding the same antigen (Figure 6). Under conditions where pre-boost T cell frequencies were approximately equivalent, the cellular immune responses induced by all three chimpanzee adenovirus vectors were boosted strongly by MVA, and there was no significant difference between the magnitudes of the post-boost responses.

In summary, all three chimpanzee adenovirus vectors elicit similar transgene product specific immune responses in the context of both single dose and heterologous prime-boost regimens.

### Optimal E4 Modification is Required to Allow Accurate Infectious Titration in the Absence of a Marker Gene

For the immunogenicity studies described above, we utilised a GFP-tagged model epitope string to facilitate titration by infectious units, which we show is required for unbiased comparisons of the immunogenicity of adenoviral vectors. However, no vaccine antigen constructs for clinical use, and indeed few pre-clinical constructs, contain a marker gene. An assay using a commercial anti-hexon antibody to identify single adenovirus-infected cells was therefore adopted [22]. This mixture of two monoclonal antibodies performs well for both group E chimpanzee adenovirus vectors and HAdV-5. However, the assay relies upon the assumption that the rate of hexon accumulation relative to transgene expression is consistent between vectors. Hexon accumulation is likely to be a function of the rate of replication in the E1 complementing cell line and hence a similar assumption is made when comparing vectors using any infectivity assay including a traditional plaque assay. Consequently there is potential for bias in the titration, if the growth rates are unequal, which could lead to inaccurate determination of doses for immunogenicity. Infectious titers of GFP expressing recombinant adenovirus vectors obtained by enumerating GFP foci were therefore compared to titers obtained using anti-hexon based assays (Figure 3C).

For HAdV-5 and ChAd63 vectors, GFP based titers were approximately twofold higher than the anti-hexon titers. However, for Y25-based vectors, the sensitivity of the anti-hexon assay varied remarkably with genetic modification of the adenovirus E4 region. No further improvement in the GFP:Hexon infectious unit ratio was observed by extending the incubation period beyond 48 hours (data not shown). For the original ChAdY25 vector which had an unmodified E4 region and exhibited poor productivity, anti-hexon titers were over 40 fold lower than GFP titers, suggesting that the rate of hexon production is considerably slower than for HAdV-5 and ChAd63 vectors. This was to be expected, given the poor yield of E4 unmodified ChAdY25 described previously. Surprisingly however, the titer of the modified ChAdY25-A vector expressing Ad5 *E4Orf6* only (the same modification as has been made to ChAd63) was still 30 fold lower by anti-hexon staining, despite a marked improvement in yield. In order to improve the transgene:hexon ratio, the E4 region was modified further, until the output of the anti-hexon immunoassay was comparable between Y25, HAdV-5 and ChAd63 based vectors (Figure 3C). The optimal E4 modified vector proposed for clinical application, ChAdY25-E, contains the *E4Orf4*, *Orf6* and *Orf6/7* coding regions from Ad5, and the *E4Orf1*, *2* and *3* coding regions from Y25 (Figure 3A). To our knowledge this is the first adenovirus vaccine vector that has been modified in this way. A Y25 based vector expressing the entire Ad5 E4 cassette showed inferior hexon accumulation to ChAdY25-E (data not shown). To determine whether the output of the anti-hexon assay was transgene dependent, a ChAdY25-E vector expressing matrix protein 1 of influenza A virus (M1) antigen was generated. To detect M1 transgene expression, a primary M1 specific monoclonal antibody (see Methods) was used in place of the commercial anti-hexon in an imunostaining assay. Figure 3D shows that the ratio of transgene gene titer to anti-hexon titer is similar for both GFP and M1 antigens.

Finally, the effect of E4 modification on immunogenicity was investigated. The immunogenicity of three different E4 modified Y25 based vectors were tested; the E4 wild type vector ChAdY25, the ChAdY25-A vector containing only Ad5 *E4Orf6* (used for the comparative immunogenicity studies described previously), and the final E4 modified ChAdY25-E vector. The three vectors were titered on GFP infectious units to abrogate differences in the readout of the hexon assay, and thus ensure accuracy of the titer. The data indicate that E4 modification has no effect on vector immunogenicity (Figure 4D).This is not surprising, since deletion of the E1 region severely reduces *in vivo* expression of adenoviral proteins by replication incompetent vectors. It does however highlight the ability to compare vectors with markedly different P:I ratios if immunisation is based on infectious titer. If the same comparison had been performed based on viral particles, the lower proportion of infectious virions in the ChAdY25 native E4 preparation would likely have manifested in lower immunogenicity, giving a misleading result.

### Seroprevalence of Vector Neutralising Antibodies in Humans

Virus neutralising antibodies acquired through natural exposure may have been responsible for limiting the potency of vectors based on common human serotypes such as HAdV-5 in recent clinical trials [4]. Adenoviruses of chimpanzee origin, including ChAd63, have previously been shown to circumvent this issue to a large extent since neutralisation titers in human sera against these viruses are generally much lower [6], [43], although in a recent Phase I clinical trial of a ChAd63-vectored malaria vaccine, there was no correlation between pre-existing anti-vector neutralisation titer and T cell responses to the encoded malaria antigen [10].

**Figure 7:**
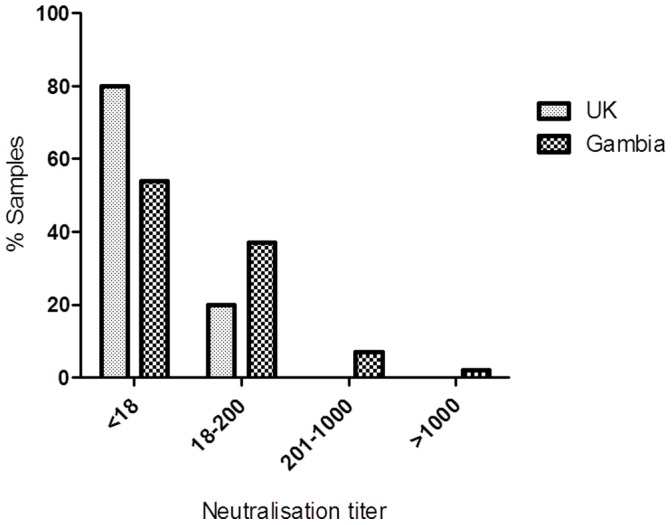
Anti-vector neutralising antibody titer in human sera from the UK (100 samples) and The Gambia (57 samples) against Y25.

It is, however, not uncommon for human sera to display some degree of neutralisation against vectors of chimpanzee origin, so it was therefore important to establish the human seroprevalence of vector neutralising antibodies against Y25. Two previously uncharacterised panels of human sera from British and Gambian adults were analysed using virus neutralisation assays against ChAdY25. The results are shown in Figure 7, and for the purposes of comparison with previous publications (see Discussion) can also be expressed as the percentage of individuals having a clinically relevant neutralizing titre (defined as a 50% neutralisation titer >200). These values are 0% for Y25 in UK adults (n = 100); and 9% for Y25 in Gambian adults (n = 57).

## Discussion

Though most adenovirus vectors have been derived using multi-copy plasmid based systems, in this study we decided to generate the molecular clone of Y25 in a bacterial artificial chromosome. This has not previously been attempted for a chimpanzee adenovirus vector, though BAC derived vectors have been described for human adenoviruses including HAdV-5 [32]. The entire chimpanzee adenovirus genome was inserted into the BAC in a single step by homologous recombination, similar to the method described by Chartier *et al.* [30], but in a manner that allowed simultaneous E1 deletion (Figure 2). The advantages of the BAC system are potentially improved genetic stability compared to multi-copy plasmids[44]; and the capacity for precise modification of the viral genome using BAC recombineering (recombination mediated genetic engineering) [15]. This technique, based on the bacteriophage Red recombination function, is highly flexible and independent of sequence specific elements such as restriction sites within the genome. The Y25 based vectors with E3 deletions and variant E4 loci could therefore be created not only rapidly and easily, but also seamlessly, such that the vector genome contained no truncated genes or unnecessary sequences. It should be noted that recombineering has been applied to multi-copy plasmid based systems but typically leads to mixed populations of modified and parental plasmids within a cell. A protracted process involving DNA extraction, dilution and retransformation is thus required to obtain a clonal population, and there are also issues with plasmid multimerisation [45], [46]. We have demonstrated that a careful consideration of vector infectivity is essential to achieve consistent and reliable immunogenicity data (Figure 4). This study, and others [7] have shown that the ratio of viral particles to infectious units significantly affects immunogenicity, and can vary over several orders of magnitude between preparations. We therefore decided to use preparations with similar P:I ratios, where possible, to minimise variability; and to base immunisation dose on infectious units, since vaccine immunogenicity correlates better with infectious dose than viral particles, as shown in Figure 4.

Infectious titer can be assessed easily and reliably by enumerating single transgene positive infected cells using a fluorescent marker such as GFP. This method was used to obtain infectious titers on which to base immunisation doses for the immunogenicity studies presented here. However, where marker genes cannot be employed, an alternative unbiased infectivity assay must be sought. We employed an immunostaining assay to identify single infected cells by detecting accumulation of hexon, the most abundant protein in the adenovirus capsid. Hexon immunostaining offers several advantages over the plaque assay, traditionally the assay of choice. Plaque assays require long incubation periods and titers are often inconsistent. Furthermore, the plaque assay is inherently insensitive for the detection of transgene-expressing virions since not all such virions induce plaque formation.

However, it is vital that the readout of any infectivity assay accurately reflects the transduction frequency of recombinant vectors. When infectious titers of GFP expressing recombinant adenovirus vectors obtained by enumerating GFP foci were compared to titers obtained using anti-hexon based assays, it was found that the output of the hexon assay was over twenty fold lower for E1/E3 deleted ChAdY25 vectors than for HAdV-5 and ChAd63 (Figure 3C). Furthermore, modification of the native Y25 E4 region to incorporate the *E4Orf6* gene from HAdV-5 was essential to increase yield of the new vector in HEK293 cells (Figure 3B), but was not sufficient to increase the output of the infectivity assay to a level comparable to HAdV-5.

We therefore created a series of vectors expressing variant E4 loci and found that the frequency of hexon positive infected 293 cells compared to transgene expressing cells was dependent on the precise nature of the E4 modification. The three chimpanzee adenovirus vectors, despite the high degree of sequence identity between them, required different genetic modifications for optimal hexon immunopositivity (equivalent to HAdV-5). While ChAd63 required only the presence of Ad5 *E4Orf6* at E4, Y25 required a chimeric E4 locus containing all six E4 open reading frames, three from HAdV-5 and three from Y25 (ChAdY25-E). Interestingly, the inclusion of Ad5 *E4Orf4* in this vector increased the frequency of hexon immunopositivity by over fourfold compared to the ChAdY25-D vector, containing the other five ORFs only (Figure 3C). The E4Orf4 protein, in complex with protein phosphatase 2A (PP-2A), has been shown to down regulate transcription from E2 and E4 adenoviral promoters and is therefore believed to form part of a negative feedback loop contributing to the temporal regulation of early viral gene expression [47]. In addition, E4Orf4-PP2A has also been shown to regulate alternative splicing of late viral transcripts which may explain the enhanced accumulation of late hexon protein [48]. We have not investigated the relevance of these potential molecular mechanisms to our results. While the addition of HAdV-5 E4Orf4 and E4Orf6/7 increased the rate of hexon production for ChAdY25-E, the relationship between this parameter and viral yield during propagation is unclear, since the addition of these proteins did not increase viral productivity in 293 cells (data not shown) under the conditions tested.

Importantly, we have also shown that vectors which exhibit sub-optimal growth or infectious titre readout without E4Orf6 replacement should not be prematurely dismissed as vaccine candidates. In many high throughput studies, chimpanzee derived adenoviruses are discarded after failing to grow in E1 complementing mammalian cell lines, often without any modification to the vector backbone at all [49]. This could ultimately limit the repertoire of serotypes tested, and cause potentially efficacious or low seroprevalance vectors to be overlooked. The BAC-based method described here will facilitate rapid E4 modification of novel vectors.

Our approach has enabled a reliable assessment of the comparative immunogenicity of Y25 based vectors with existing chimpanzee adenovirus vectors ChAd63 and AdC68. ChAd63 recently became the first chimpanzee adenovirus vector to enter clinical trials and has elicited unprecedented frequencies of antigen-specific T cells in humans when boosted by MVA [10]. It has since been used in several malaria vaccine trials, consistently delivering potent cellular and humoral immune responses [11]. AdC68 was the first E1 deleted chimpanzee adenovirus vector to be created [5] and has since been demonstrated to be a highly efficacious vaccine vector in pre clinical vaccine studies [50]. Transgene product specific cellular immunogenicity of ChAdY25, in both single shot and prime boost regimens, was equivalent to both ChAd63 and AdC68 vectors (Figure 5 and 6). That chimpanzee adenovirus vectors of the species *Human adenovirus E* elicit cellular immune responses of very similar magnitudes is in agreement with some previous studies [9], [51], but not others [7], [8].

It has previously been demonstrated that chimpanzee adenovirus vectors, including ChAd63, are less susceptible to neutralising antibodies in humans than vectors based on common human adenovirus serotypes such as HAdV-5 [6], [43], [51]. In a recent clinical trial assessing efficacy of a HAdV-5 vector expressing HIV-1 antigens, T cell immunogenicity was significantly lower in individuals with a pre existing anti-HAdV-5 neutralisation titer of >200 [4]. In a previous study by Dudareva *et al*, the percentage of serum samples exhibiting 50% ChAd63 neutralizing titres >200 was just 4% in a juvenile Kenyan population [6] and ChAd63 was significantly less seroprevalent than HAdV-5 (4% ChAd63, 23% HAdV-5), in common with other chimpanzee adenoviruses [6], [43], [51], [52]. We found that neutralisation titers >200 against ChAdY25 were also particularly low in the UK (0%) and Gambian (9%) sera tested (Figure 7). Analysis of larger numbers of samples will be needed to gain a better understanding of the effect of age, geography, or other factors upon adenoviral seroprevalence. Whether differences in seroprevalence may significantly affect the clinical utility of the vectors, remains to be fully elucidated: interestingly, in a recent study of ChAd63 immunogenicity there was no impact of anti-vector antibodies on T cell immunogenicity to the encoded malaria antigen [10]. Sera from human individuals vaccinated with the ChAd63 vector did not neutralise Y25 despite developing extremely high neutralization titers against ChAd63. This confirms that the two vectors are serologically distinct, and suggests that they may be able to be used in concurrent vaccine programs without a reduction in efficacy.

We have determined, in a carefully controlled system, that species E chimpanzee adenoviruses are equivalent terms of their potential to elicit immune responses. T cell responses against both the transgene product and the vector virion were almost identical between the three vectors tested. This is not surprising, given the strong phylogenetic relationship between them. Nevertheless, this study has shown that expanding the repertoire of vector serotypes available, and testing them in the manner described, may prove advantageous since we have demonstrated a difference in human seroprevalence between vectors that share a high degree of genetic identity. The low seroprevalence of vector neutralising antibodies against Y25 suggests that new vectors based on this virus are likely to be efficacious in a clinical setting. To this end, vector ChAdY25-E has been renamed ChAdOX1 for use in forthcoming clinical trials.

## Supporting Information

Table S1 Primers used in the construction of ChAdY25-A to –E.
(DOCX)
