# Supplementary material for: A Novel Chimpanzee Adenovirus Vector with Low Human Seroprevalence: Improved Systems for Vector Derivation and Comparative Immunogenicity
Source: PLoS One. 2012 Jul 13;7(7):e40385. doi: 10.1371/journal.pone.0040385 (PMC3396660; doi:10.1371/journal.pone.0040385)
Supplement: Table S1 — Primers used in the construction of ChAdY25-A to –E. (DOCX) [file pone.0040385.s001.docx]

| **Use** | **Primer Name** | **Template** | **Sequence 5’- 3’** |
| --- | --- | --- | --- |
| Amplification of LFI homology arm of rescue vector | LFI Fwd | Y25 gDNA | GCGATCGCGTTTAAACCCATCATCAATAATATACCTCAA |
|  | LFI Rev |  | CCTGCAGGCTTTCAAAGTGTAGATCTGACTCG |
| Amplification of LFII homology arm of rescue vector | LFII Fwd | Y25 gDNA | CCTGCAGGAGTGAGTAGTGTTCTGGGGCGGGGGAGGAC |
|  | LFII Rev |  | GAATTCCGGTAACATCGCCAATCTCAAG |
| Amplification of RF homology arm of rescue vector | RF Fwd | Y25 gDNA | GAATTCGCAATTTTTAAGAAAATCAACA |
|  | RF Rev |  | GCGGCCGCGTTTAAACCATCATTCAAATATATACCTCAAAC |
| Replacement of Y25 E3 region with *GalK* adding unique *PacI* site. | E3del LH | *GalK* cassette | *TCATCCCGAACTTTGACGCCATCAGCGAGTCGGTGGACGGCTACGATTGA***TTAATTAA**CCTGTTGACAATTAATCATCGGCA |
|  | E3del RH |  | *CATTTTTAATCTTTATTTTTACTGGAGGGTAAAGGGGTAGGGGGTTAGTTGA***TTAATTAA**TCAGCACTGTCCTGCTCCTT |
| Replacement of native Y25 E4 region with *GalK* | E4delLH | *GalK* cassette | *CTGAAGCAGAAAAAAATAAAGTTCAAGTGTTTTATTGATTCAACAGTTTTC***CGGACCG**CCTGTTGACAATTAATCATCGGCA |
|  | E4 wholedel RH |  | *TAGAGCTCAGCCTTTTCTCTGACTCTTGCGCCTGCCGTGCTCGGTAAGCT***CGGACCG**TCAGCACTGTCCTGCTCCTT |
|  | E4Orf4del RH |  | *CGGGACCTGCATTTTGAAGTGCTCCGAGACCGTTTGGAATAAAGTTAATC***CGGACCG**TCAGCACTGTCCTGCTCCTT |
| Replacement of *GalK* with variants of HAdV-5 E4 region | Ad5E4Orf6insLH | pAdPL-DEST | *CTGAAGCAGAAAAAAATAAAGTTCAAGTGTTTTATTGATTCAACAGTTTTC*ctacatgggggtagagtcataatcg |
|  | Ad5E4Orf6/7insLH | pAdPL-DEST | *CTGAAGCAGAAAAAAATAAAGTTCAAGTGTTTTATTGATTCAACAGTTTTC*tcacagaaccctagtattcaacctg |
|  | Ad5insE4Orf4RH | pAdPL-DEST | *CGGGACCTGCATTTTGAAGTGCTCCGAGACCGTTTGGAATAAAGTTAATC*atgactacgtccggcgttccatttgG |
|  | Ad5insE4wholedelRH | pAdPL-DEST | *TAGAGCTCAGCCTTTTCTCTGACTCTTGCGCCTGCCGTGCTCGGTAAGCT*atgactacgtccggcgttccatttgG |
|  | Ad5Orf4insOrf4delRH | pAdPL-DEST | *CGGGACCTGCATTTTGAAGTGCTCCGAGACCGTTTGGAATAAAGTTAATC*atggttcttccagctcttcccgctc |

**Table S1: Primers used in the construction of ChAdY25-A to –E**

Key: Restriction enzyme sites **bold**; 50 base pair homology regions for recombineering shown in *italic*
